# Myocardial regeneration by transplantation of modified endothelial progenitor cells expressing SDF-1 in a rat model

**DOI:** 10.1111/j.1582-4934.2012.01539.x

**Published:** 2012-09-26

**Authors:** Alexander Schuh, Andreas Kroh, Simone Konschalla, Elisa A Liehn, Radoslav M Sobota, Erik AL Biessen, Ilze Bot, Tolga Taha Sönmez, Andreas Schober, Nikolaus Marx, Christian Weber, Alexander Sasse

**Affiliations:** a Department of Cardiology Pulmonology and Vascular Medicine Medical Faculty, RWTH Aachen UniversityGermany; b Institute for Molecular Cardiovascular Research (IMCAR), RWTH Aachen UniversityGermany; c Department of Biochemistry and Molecular Biology Center for Experimental Bioinformatics, University of Southern DenmarkOdense; d Department of Pathology Cardiovascular Research Institute Maastricht (CARIM), Maastricht University Medical CenterThe Netherlands; e Division of Biopharmaceutics Leiden/Amsterdam Center for Drug Research, Leiden UniversityThe Netherlands; f Department of Oral and Maxillofacial Surgery, RWTH Aachen UniversityGermany; g Institute for Cardiovascular Prevention, Ludwig-Maximilians-University (LMU) MunichMunich, Germany; h Munich Heart Alliance, Ludwig-Maximilians-University (LMU) MunichMunich, Germany

**Keywords:** myocardial infarction, cell transplantation, endothelial progenitor cells, chemokines, heart regeneration

## Abstract

Cell based therapy has been shown to attenuate myocardial dysfunction after myocardial infarction (MI) in different acute and chronic animal models. It has been further shown that stromal-cell derived factor-1α (SDF-1α) facilitates proliferation and migration of endogenous progenitor cells into injured tissue. The aim of the present study was to investigate the role of exogenously applied and endogenously mobilized cells in a regenerative strategy for MI therapy. Lentivirally SDF-1α-infected endothelial progenitor cells (EPCs) were injected after 90 min. of ligation and reperfusion of the left anterior descending artery (LAD) intramyocardial and intracoronary using a new rodent catheter system. Eight weeks after transplantation, echocardiography and isolated heart studies revealed a significant improvement of LV function after intramyocardial application of lentiviral with SDF-1 infected EPCs compared to medium control. Intracoronary application of cells did not lead to significant differences compared to medium injected control hearts. Histology showed a significantly elevated rate of apoptotic cells and augmented proliferation after transplantation of EPCs and EPCs + SDF-1α in infarcted myocardium. In addition, a significant increased density of CD31^+^ vessel structures, a lower collagen content and higher numbers of inflammatory cells after transplantation of SDF-1 transgenic cells were detectable. Intramyocardial application of lentiviral-infected EPCs is associated with a significant improvement of myocardial function after infarction, in contrast to an intracoronary application. Histological results revealed a significant augmentation of neovascularization, lower collagen content, higher numbers of inflammatory cells and remarkable alterations of apoptotic/proliferative processes in infarcted areas after cell transplantation.

## Introduction

Cell transplantation as well as gene therapies are promising strategies in the treatment of cardiac dysfunction after MI. Several studies demonstrated improvement of myocardial function after transplantation of various cell types [1–7]. Recently, we and other groups could demonstrate that different cell types act *via* different mechanisms to improve myocardial contractility [5–8]. One important point seems to be the amount of vessel structures in infarcted areas. Evidence for a significantly higher amount of vessels in infarcted areas pointed towards neovascularization being responsible for a better contractile function [5].

However, because most implanted cells were lost to ischaemia, apoptosis and inflammatory alterations, the aim of this study was to improve the function of remaining transplanted cells to augment neovascularization and nutrition of damaged myocardium. Cell engineering is another possibility to optimize cellular function after transplantation. Nakamura *et al*. could demonstrate a significant improvement of cell survival and ventricular function after infection of transplanted cells by pre-treatment with antiapoptotic Bcl-2 gene [9]. Additional studies dealing with infection of cells with different growth factors could underline, that genetical alterations could improve the effectiveness of cell transplantation. Yang *et al*. established a method to transfect vascular endothelial factor (VEGF) gene into mesenchymal stem cells and showed a better restoration of heart function and angiogenesis after MI [10]. Even the SDF-1 is shown to be an effective and important chemokine inducing neovascularization even in infarcted areas [11]. The production of SDF-1 is transiently increased after tissue ischaemia and initiates the migration of progenitor cells. Furthermore, it has a major part in embryological hemato-, vasculo- and cardiogenesis.

In the present study we transplanted cells lentivirally infected with the chemokine SDF-1 to improve neovascularization by augmented and prolonged SDF-1 production after cell transplantation as well as to mobilize host EPC to the infarcted areas. The aim of our study was to combine an exogenous and endogenous cell therapy.

Another goal of the study presented herein was to determine, if an intracoronary application in a rat model is feasible and comparable to an intramyocardial injection of transplanted cells. Furthermore, we chose an ischaemia/reperfusion model (90 min. of ischaemia) to simulate the case of a MI, followed by a catheterization as soon as possible in a rodent model, expecting to transfer these methods to large animal or in the future to human studies. Despite all various cell transplantation studies the optimal cell type and delivery technique further remains to be determined. Therefore we used a special new rodent transcarotid balloon catheter system for the intracoronary application of cells.

## Materials and methods

### Cells

**Fig 1:**
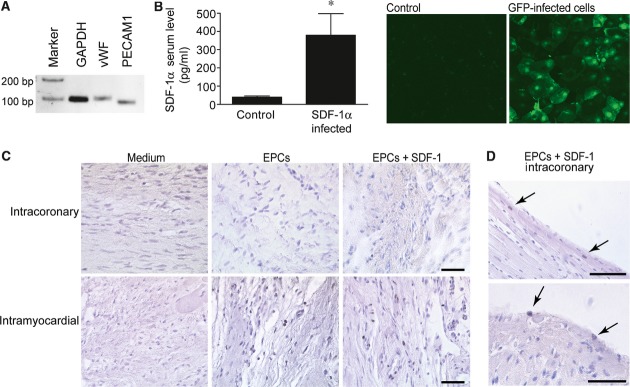
Infected cells and detection of transplanted cells in infarcted areas. (A) Isolated and cultured EPC were characterized by mRNA isolation and RT-PCR of endothelial markers (von Willebrand Factor–vWF- and PECAM1). (B) SDF-1 serum levels are significantly higher after lentiviral infection compared to control levels (*P < 0.05 *versus* control, left). Control EPCs infections using virus carrying cDNA encoding GFP have shown at least 90% efficiency (green staining for GFP, right). (C) Immunohistochemical BrdU staining of infarcted areas after cell transplantation shows positive signals for BrDU-labelled EPC-derived cells (see arrows), *e.g*. in capillary-like structures. Objective 40×, scale bars: 25 μm.

The EPCs were isolated from rat spleens, which were removed from female adult Sprague-Dawley rats (200–250 g). After mincing spleens into small pieces mononuclear cells (MCs) were separated by repeated pipetting and Biocoll (Biochrom AG, Berlin, Germany) density gradient centrifugation, washed twice in PBS and counted. Finally, the MCs were resuspended in microvascular endothelial growth medium MV2 (PromoCell, Heidelberg, Germany) and plated in a T-25 culture flask coated with 10 μg/ml human fibronectin (Harbor Bioproducts Inc., Indianapolis, IN, USA). After four days non-adherent cells were removed and fresh medium was added. At day seven, adherent cells were detached with trypsin, counted, resuspended at 10^6^ cells/100 μl PBS and transplantated. On the day before transplantation, cells were incubated with BrdU (Zymed, Wien, Austria) as described by manufacturer. EPC were characterized by mRNA isolation and RT-PCR of endothelial markers (von Willebrand Factor- vWF- and PECAM1, Fig. 1A). Briefly, mRNA was isolated from the EPC after seven days in culture using Micro-mRNA Isolation Kit (Qiagen, Hilden, Germany) and RT-PCR was performed with Omniscript kit (Qiagen). Following oligonucleotide primers specific were used for rat vWF cDNA (forward primer: 5′-AAGCTCTGGGTAAAGACACTGC-3′ and reverse primer: 5′-CACCAGAGAGTTCAGCTCCTTT-3′) and PECAM1 cDNA (forward primer: 5′-TACGTCACTGTGCAGGAGTTCT-3′ and reverse primer: 5′-GCAGCTGGTTTCCTTCTATGAT-3′). Rat GAPDH cDNA served as housekeeping gene and was amplified in parallel with the gene of interest (forward primer: 5′-CTGTGACTTCAACAGCAACTCC-3′, and reverse primer: 5′-TACCAGGAAATGAGCTTCACAA-3′).

### Lentiviral infection of cells

The HEK 239T cells were cultured in DMEM containing 4,5 g/l glucose, 10% foetal calf serum (FCS), 2 mMol/l L-glutamine, 100 U/ml penicillin and 100 μg/ml streptomycin (PAA, Cölbe, Germany). The SDF-1α expression vector (pRRl-cPPt-CMV.SDF-1α: LV.SDF-1α) was constructed by ligation of SDF-1α cDNA into SMA1 digested pRRl-cPPt-CMV-PreSIN [12–14]. An expression vector containing the GFP cDNA insert was constructed as a positive control and both expression plasmids were sequence verified before use. Lentivirus (LV) expression and helper vectors were cotransfected into HEK293T cells by the calcium phosphate method, medium collected at 24 and 48 hrs and virus titres determined as described by Bot *et al*. [14]. Viral integrants were determined by qPCR analysis (forward primer: GTGCAGCAGCAGAACAATTTG, reverse primer: CCCCAGACTGTGAG TTGCAA). Virus stocks were aliquotted and stored at −20°C until further use. Prior viral infection EPCs were grown in Microvascular endothelial growth medium MV2 (PromoCell). On the day of infection cells were rinsed with PBS and medium was changed to DMEM with 10% FCS. Infection was performed in presence of 10 μg/ml diethylaminoethyl dextran at 30°C. After 24 hr, cells were washed with PBS and were resuspended again in MV2 medium. Control infections with virus carrying cDNA encoding GFP have shown at least 90% efficiency. SDF-1 concentration in cultured cells was evaluated by ELISA, which was performed from serum or cell medium with Quantikine Mouse CXCL12/SDF-1 Immunoassay (R&D Systems, Wiesbaden-Nordenstadt, Germany). Cells were transplanted 3 days after infection with LV.SDF-1α.

### Generation of myocardial infarctions and cell transplantation

Female adult Sprague-Dawley rats (200–250 g) were incubated under general anaesthesia (1 ml/kg ketamine and 10 mg/kg xylasine, intraperitoneal) and positive pressure ventilation was maintained (room air supplemented with oxygen) using a rodent respirator. Hearts were exposed through a 2-cm left thoracotomy and temporal myocardial ischaemia leading to MI was induced by suture occlusion of the LAD between the left atrium and the right pulmonary outflow tract (7/0 polyprolene, Ethicons). A 90 min. ischaemia regimen was chosen as it resulted in acceptable intraprocedural death and significant loss of myocardial function. After 90 min. of ligation/reperfusion of the LAD BrdU-labelled SDF-1α infected EPCs (10^6^, *n* = 8) were injected into the myocardium or intracoronarily, respectively (*n* = 8). In additional animal groups we injected non-transduced EPCs (intramyocardial (*n* = 8) and intracoronary (*n* = 8)) and medium as control group (*n* = 10 per group). The areas for injection were chosen by visual identification based on changes of appearance due to temporal ischaemia and wall motion akinesis. Cells were transplanted into marginal zones of the MI by syringe injection (for 1-min. injection time) at three distinct but adjacent sites. After injection, the puncture holes were closed by suture, which served as a marker for the area of transplantation at follow-up thoracotomy. Intracoronary application of cells was performed by a special novel rodent catheter system that was developed in cooperation with Vimecon (Herzogenrath, Germany) for this study. After preparation of the left carotid artery the catheter system was inserted through the carotid artery and advanced to the ascending aorta, which was blocked above the coronary ostia by the inflated balloon catheter for 60 sec. During these 60 sec. cells or control medium was injected into the coronary arteries from the tip of the catheter. After reperfusion and cell application muscle layer and skin incision were closed with silk sutures. Animal experiments were approved by local authorities and complied with German animal protection law.

### Echocardiography

Eight weeks after MI and TX (*i.e*. prior to transplantation) rats were anaesthetized and two-dimensional and M-mode measurements were performed with a SONOS 7500 (Philips, Hamburg, Germany) with a 15-MHz linear phased-array probe. The animals were anaesthetized and placed in the supine or lateral position. Parasternal long-axis and short-axis views were obtained, ensuring that the mitral and aortic valves and apex were well visualized. Fractional shortening was measured in long axis and orthogonally in the short axis, average of both results was used for additional analysis.

### Langendorff perfusion and assessment of the infarct size

Eight weeks after MI and TX the rats were anaesthetized and hearts removed. Heart function was analysed applying a Langendorff apparatus with filtered Krebs-Henseleit buffer at a pressure of 100 mmHg equilibrated with 5% CO_2_ and 95% O_2_. A latex balloon was placed into the left ventricle to measure pressure and heart rate, using a commercially available isolated heart apparatus (Hugo Sachs Electronik, March-Hugstetten, Germany). Perfusion pressure, LV developed pressure (LVDP; the difference between minimal diastolic pressure and maximal systolic pressure), coronary flow and heart rate were measured continuously (the last two did not show any statistically significant variance throughout the experiments). After 20 min. of stabilization, baseline values were recorded. Then preload was increased from 5 mmHg to 10, 15 and 20 mmHg. LVDP was recorded at the different preloads and developed pressure was calculated. The scar size of Left ventricle-free wall was assessed by computed planimetry of digitized images taken from hearts fixed in distension (30 mmHg) with 10% formalin and cut into 5 μm slices.

### Identification of the cells and analysis of the infarction area

For cell identification, slides (5 μm) were stained with anti-BrdU kit (Zymed). Universal quick kit (Vector Laboratories, Axxora, Loerrach, Germany) were used with anti-CD31 antibody (Santa Cruz Biotechnology, Heidelberg, Germany) for vessel identification. For SDF-1 staining, an anti-SDF-1 antibody (Serotec, Düsseldorf, Germany) was used followed by a secondary, FITC-conjugated antibody. The nuclei undergoing apoptosis were stained with MEBSTAIN apoptosis kit II (MBL, Woburn, MA, USA). Accustain trichrome stain (Masson; Sigma-Aldrich, Seelze, Germany) was used to determine collagen content of infarcted regions. The stained areas were measured by computer-assisted planimetry (Diskus software, Hilgers). Macrophages and Neutrophils were stained with alpha-naphthyl acetate esterase (Sigma-Aldrich) and naphthol AS-D chloroacetate (Sigma-Aldrich) respectively. To evidence proliferation, the sections were stained for Ki-67 (DAKO, Hamburg, Germany), followed by an anti rat-Cy3 antibody.

### Data analysis

Data represent mean ± S.D. Statistical analysis was performed with Prism 4 and 5 software (Graph Pad) using unpaired Student *t*-test or 1-way anova followed by Newman–Keuls test. Differences with *P* < 0.05 were considered significant.

## Results

### Transfection efficacy and tracking of EPC after transplantation

Control infection experiments of EPCs performed with lentivirus (LV) carrying cDNA encoding GFP 72 hrs after infection demonstrated an average efficiency of 90%, (Fig. 1A). LV-SDF1 infected EPCs efficiently secreted SDF-1 into the cell culture medium. Medium SDF-1 levels compared to control medium of non-infected EPCs were highly significantly elevated (LV-SDF-1 infected cells 379.4 ± 118 pg/ml *versus* 39.4 ± 6.7 pg/ml for non-infected EPC, *P* < 0.05, Fig. 1B).

Pre-implantation EPC that had been BrdU-labelled, 8 weeks after transplantation of EPCs were detected both at the injection site as well as incorporated in capillary-like structures after intracoronary application (Fig. 1C). Unfortunately, the Brd-U signals were very rare and it was impossible to quantify the number of injected cells refined in myocardium after the transplantation.

### Remodelling and inflammation in infarcted areas

**Fig 2:**
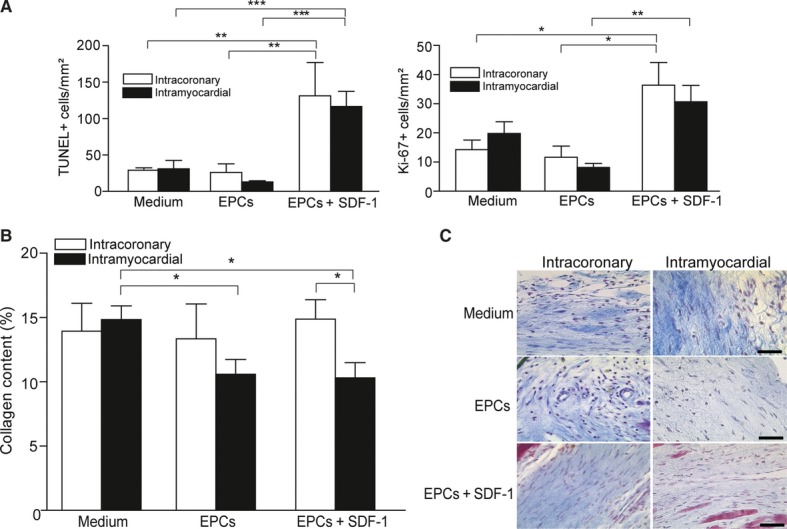
Apoptosis, proliferation and collagen content in infarcted areas. TUNEL staining was used to identify increased numbers of apoptotic cells (A) in with SDF-1 transfected cells treated hearts (*P < 0.05 *versus* control). (B) To assess proliferation, the sections were stained for Ki-67 (DAKO), followed by an anti-rat-Cy3 antibody (green; *P < 0.05 *versus* control). Collagen content in infarcted areas (C, D) was significantly lower after intramyocardial transplantation of EPC and lentivirally with SDF-1 transfected EPC. Intracoronary cell application did not result in changes of collagen concentration (blue; scale bar: 100 μm; *P < 0.05 *versus* control).

Apoptosis measured by TUNEL staining increased in the infarcted area after transplantation of SDF-1 expressing EPC, independent of their application mode (intramyocardial: LV-SDF-1-EPC TX 116.5 ± 20.7 TUNEL^+^ cells/mm^2^ *versus* control TX 31.7 ± 11.5 TUNEL^+^ cells/mm^2^; *P* < 0.05, intracoronary: LV-SDF-1-EPC TX 131.3 ± 45.6 TUNEL^+^ cells/mm^2^ *versus* control TX 19.4 ± 2.2 TUNEL^+^ cells/mm^2^; *P* < 0.05). When EPC only were injected into myocardium or *via* the coronary circulation the degree of apoptosis did not differ from medium controls (Fig. 2A).

A similar pattern is detected with the assessment of cellular proliferation in the infarcted myocardium. In both groups of EPC plus SDF-1 an augmented proliferation in the infarcted area after the application is measured (intramyocardial: LV-SDF-1-EPC TX 30.7 ± 5.6 Ki-67^+^ cells/mm² *versus* EPC 19.8 ± 4 Ki-67^+^ cells/mm²; *P* < 0.05, intracoronary: LV-SDF-1-EPC TX 36.4 ± 4.5 Ki-67^+^ cells/mm² *versus* control TX 14.2 ± 3.4 Ki-67^+^ cells/mm²; *P* < 0.05, Fig. 2A). EPC alone – independent of application route – did not significantly affect the proliferation compared to medium injected controls (Fig. 2B).

Interestingly the collagen content in the infarcted area measured by specific staining was significantly lower after direct intramyocardial but not after intracoronary EPC transfer when compared to the untreated control (Fig. 2B and C). The additional expression of SDF-1 did not alter the collagen content (intramyocardial: EPC TX 10.6 ± 1.2% of infarcted area *versus* LV-SDF-1-EPC TX 10.3 ± 1.2% of infarcted area *versus* control TX 14.8 ± 1.1% of infarcted area, *P* < 0.05, Fig. 2B). The infarct size after MI and cell or control medium injection did not differ between the groups (data not shown).

### Angiogenesis and cellular inflammatory response

**Fig 3:**
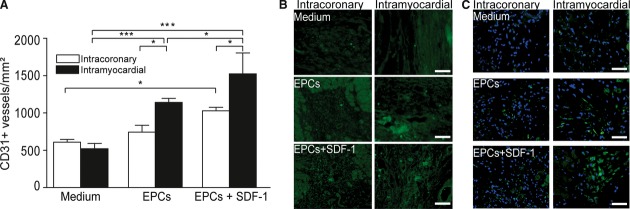
Vessel density and SDF-1 expression in infarcted areas. (A, B) Vessel density per mm^2^ (green: CD 31 positive vessels; objective 20×; scale bar: 50 μm; *P < 0.01 *versus* control) was significantly increased in cell-treated hearts compared with controls. Transplantation of SDF-1 transfected cells further increased vessel density (*P < 0.05; **P < 0.01; ***P < 0.001), (C) but also the SDF-1 expression in infarcted hearts (green: SDF-1 positive staining; blue: DAPI nucleus staining; objective 40×; scale bar: 25 μm).

The effects on angiogenesis and vascular density were assessed by staining and quantification of CD31-positive vessels in the area of infarction. First looking at EPC injection alone intracoronary EPC application did not increase vessel density whereas intramyocardial injection led to quite a significant increase (intracoronary: EPC TX 744 ± 92 vessels/mm² *versus* control TX 609 ± 38 vessels/mm²; p ns. intramyocardial: EPC TX 1142 ± 53.6 vessels/mm² *versus* control TX 520 ± 72.3 vessels/mm²¸ *P* < 0.001. Difference between i.c. and i.m. *P* < 0.05). EPCs enhanced with SDF-1 infection induced a more pronounced albeit differentiated angiogenesis. SDF-1 expressing EPC injected i.c. induced a significant increase of angiogenesis (LV-SDF-1-EPC TX 1024 ± 47 vessels/mm²) compared to control (*P* < 0.05) although not reaching significance compared to the EPC only group. Intramyocardial injection of LV-SDF-1-EPC induced the most prominent angiogenesis response almost tripling the amount of blood vessels found in controls (LV-SDF-1-EPC TX 1523 ± 280 vessels/mm²) meeting statistical significance in comparison to EPC i.m. (*P* < 0.05) and medium controls (*P* < 0.001) but even in comparison to i.c. injection of the same cell type (*P* < 0.01, Fig. 3A and B). As expected, we found an increased SDF-1 staining in the transfected EPC groups, correlating successfully to CD31 staining (Fig. 3C).

As indicators of inflammation, we assessed monocyte/neutrophil cell infiltration in all groups by staining for specific/unspecific esterase. The most evident infiltration of monocytes within the infarcted territory was seen in the EPC plus SDF1 groups (intramyocardial: LV-SDF-1-EPC TX 182.8 ± 40.7 cells/mm^2^ *versus* control TX 47.1 ± 3 cells/mm^2^, *P* < 0.01; intracoronary: EPC/SDF-1 TX 146.8 ± 45.4 cells/mm^2^ *versus* control TX 57.1 ± 11 cells/mm^2^, ns). When EPC alone were injected again only the intramyocardial group reached significance compared to controls (intramyocardial: EPC TX 93.1 ± 7.4 cells/mm^2^, *P* < 0.05 *versus* controls; intracoronary: EPC TX 55.9 ± 16.8 cells/mm^2^, ns) and in spite of a trend seen in the absolute numbers the monocyte infiltration in the EPC groups did not differ from LV-SDF-1-EPC groups. Moreover, being the main source of producing proteases, the number of monocytes/macrophages correlates to collagen content.

**Fig 4:**
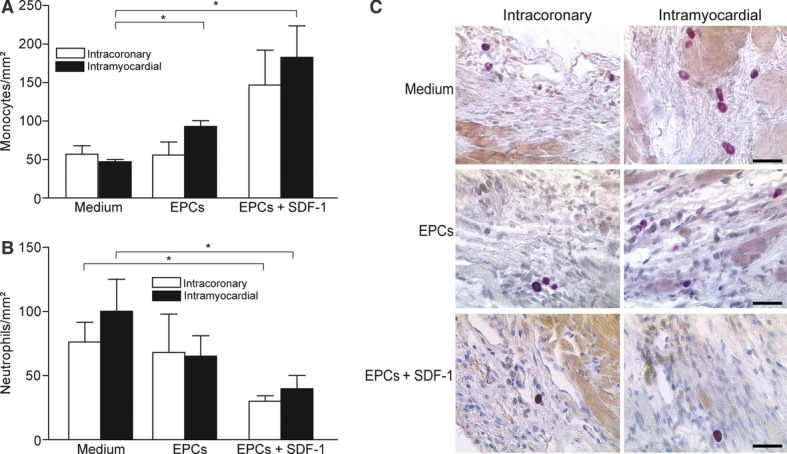
Cellular inflammatory response in infarcted areas. (A, C) Number of monocytes (yellow-brown) were increased in infarcted areas after direct intramyocardial injection and after intracoronary application but particularly when cells were also expressing SDF-1, while neutrophils (B,C) were decreased in cell-treated hearts (red; objective 40×; scale bar: 25 μm; *P < 0.05 *versus* control).

In sharp contrast, neutrophil influx was impaired after transplantation of LV-SDF-1-EPC but not after EPC only transfer – independent of application type (intramyocardial: EPC TX 65.2 ± 15.9 cells/mm^2^ *versus* LV-SDF-1-EPC TX 39.8 ± 10.4 cells/mm^2^ *versus* control TX 100.2 ± 25 cells/mm^2^, intracoronary: EPC TX 68.1 ± 29.9 cells/mm^2^ *versus* LV-SDF-1-EPC TX 30.1 ± 4.2 cells/mm^2^ *versus* control TX 76.2 ± 15.4 cells/mm^2^, *P* < 0.05; Fig. 4).

### Cardiac function

**Fig 5:**
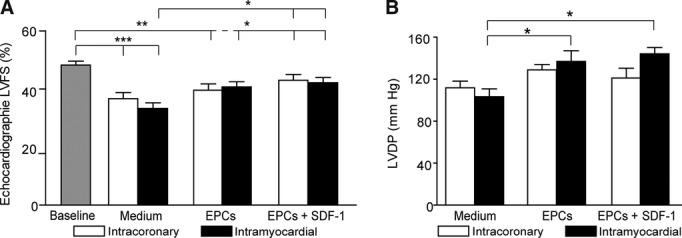
Echocardiographic results and isolated heart studies. LV function as measured per fractional shortening in% (A) after transplantation was significantly improved after intramyocardial transplantation of EPCs/lentivirally with SDF-1 infected EPCs (*P < 0.05; **P < 0.01; ***P < 0.001). Isolated heart studies (B): Significantly higher base line pressures (*P < 0.05) after intramyocardial transplantation of EPCs/lentivirally with SDF-1 infected EPCs.

Although we were able to demonstrate a differentiated tissue response the key performance indicator of the heart is the assessment of its contractile function. To assess cardiac function, we performed two-dimensional echocardiography (fractional shortening (FS) in %) before and after MI/cell transplantation (Fig. 5A). Eight weeks after MI echocardiographic analysis revealed a significantly impaired LV function in the medium control groups (baseline FS: 48.3 ± 5.9%, medium control i.c.: 36.7 ± 6.8% and medium control i.m.: 33.3 ± 5.9%; *P* < 0.001 compared to baseline; ns between controls). The LV FS deteriorated by 11.6% and 14.9% respectively. Transplantation of EPC improved LV FS by 2.95% comparing intracoronary application (EPC i.c. FS: 39.6 ± 6.0%; ns compared to medium control i.c.). In the intramyocardial application group the FS increased by 7.4% compared to medium control group but again not reaching statistical significance (EPC i.m. FS: 40.7 ± 5.0%; ns compared to medium control i.m.). Transplantation of SDF-1 infected EPC led to a LV FS increase of 6.4% compared to controls (LV-SDF-1-EPC i.c. 43.0 ± 5.7%; ns compared to medium control i.c.) while the increase seen with i.m. application of LV-SDF-1-EPC reached statistical significance with an increase of LV FS by 8.9% (LV-SDF-1-EPC i.m. 42.2 ± 5.1%; *P* < 0.05 compared to medium control i.m.). Although the LV-SDF-1-EPC transplanted hearts showed a trend towards better contractility there was no significant difference between EPC and EPC/SDF-1 groups regarding this parameter.

For further quantification of LV function, we performed isolated heart studies with retrograde perfusion according to Langendorff. LVDP was significantly augmented in hearts after intramyocardial transplantation of EPCs or SDF-1 infected EPCs (LVDP: after EPC TX 137 ± 24.9 mmHg *versus* LV-SDF-1-EPC 144.1 ± 16.2 mmHg *versus* control 103.4 ± 19.2 mmHg, *P* < 0.05). Intracoronary injection showed a trend towards improved LVDP but did not reach statistical significance (LVDP: after EPC-TX 128.9 ± 14.7 mmHg *versus* LV-SDF-1-EPC 121.1 ± 24.5 mmHg *versus* control 111.8 ± 19.4 mmHg, *P* < 0.05, Fig. 5B).

## Discussion

Recent studies have shown that cardiomyocyte regeneration is a process taking place throughput our entire life, at the age of 55 45% of all cardiomyocytes have been generated after adolescence and 0.45–1% are regenerated every year [15]. Cell therapy for cardiac repair has been propagated not only in various permutations in animal models but also clinical trials. Especially the ltater mostly used bone marrow-derived mononuclear cells (BMCs) with meta-analyses of these trials demonstrating an average improvement of LV ejection fraction around 4% [16, 17]. Other cell populations that have been studied are haematopoetic and mesenchymal progenitor cells. Recent interest has also been focussed on resident cardiac progenitor cells. These resident cells are expected to differentiate into contractile cardiomyocytes although the other types of cells are thought to be more involved in influencing the myocardial environment facilitating remodelling, angiogenesis and inflammatory response [18].

On the other hand gene therapy of the heart is a promising approach to interfere with pathophysiological processes. However it has been limited by problems of delivery, efficacy, cell specificity and side effects [19]. Experimental animal studies have shown to promote angiogenesis and attenuate apoptosis. A recently published study analysed the response to intramyocardial injection of adenoviral vectors encoding SDF-1 in an animal model of MI demonstrating changes in collagen expression as well as TGF-β1, TIMP-1 and TIMP-2 [20]. Gene therapy appears to be an ideal partner of cell therapy allowing specific alteration of transplanted cells, using the cells as a vehicle and limiting systemic side effects. Rong *et al*. demonstrated that transplantation of heart cells overexpressing human growth hormone led to induced angiogenesis and diminished apoptosis [21]. Also combined growth hormone gene transfer and cell transplantation provided an effective strategy for improving post-infarction ventricular function. To transfect cells with different hormones, cytokines and growth factors several techniques have been established [22–25]. Genetically altered cells or cells pre-conditioned with growth factors seem to have an additional impact on improvement of myocardial function [26–28]. Yang *et al*. could show in a rat model that transplantation of mesenchymal stem cells (MSCs), liposomally transfected with VEGF gene ameliorated myocardial perfusion and restored heart function more potently than either cellular or gene therapy alone [10]. In another study by Liu *et al*. [29] mesenchymal stem cells were transfected by an adenoviral vector with angiogenin, the angiogenin modified stem cells then enhanced the tolerance of engrafted cells to hypoxia injury *in vitro* and improved their viability in infarcted hearts, thus helping preserve the LV contractile function and attenuate LV remodelling through vasculogenesis. Sun *et al*. [30] used human angiopoietin-1 (hAng1)-modified MSCs to treat acute MI in rats. The hAng1 gene was transfected into cultured rat MSCs also using an adenoviral vector. The results indicated that hAng1-modified MSCs improved heart function and angiogenic effects. Despite of the limitations shown in animal and clinical studies cell and gene therapies remain potential new treatments for cardiovascular diseases [30].

In most studies performed with BMCs neovascularization was the key mechanism to achieve cardiac repair after MI while direct cardiomyogenesis is not likely to be expected from this strategy [8]. Inflammatory processes triggered by cell transplantation potentially contribute to and modify remodelling processes [6]. Modulation of inflammatory processes as well as paracrine effects by transplanted cells have also been suggested to play an important role in maintaining cardiac function. Dai Y *et al*. [28] concluded that mobilized progenitor cells are highly cytoprotective and carry protective genes responsible for cardiac repair.

The CXC chemokine SDF-1 has been shown to play a major role in embryological hemato-, vasculo- and cardiogenesis and is transiently increased after tissue ischaemia and initiates the migration of progenitor cells. Recently [5], we could demonstrate that injection of SDF-1 alone and combined with EPC in a MI model is able to augment neovascularization and seems to be associated with attenuated heart function. Also Elmadbouh *et al*. [11] transfected human stromal cell derived factor (SDF-1) gene into skeletal myoblasts and transplanted these cells to establish a transient SDF-1 gradient to favour extra-cardiac stem cell translocation into infarcted heart. They could show that *ex vivo* SDF-1 transgene delivery promotes stem and progenitor cell migration to the heart and enhances angiomyogenesis in the infarcted areas. Recently it has also been demonstrated that SDF-1 expression in the heart enhances the homing of bone marrow derived stem cells to the area of infarction [31].

The aim of the present study was to combine exogenous stem cell therapy with enhanced mobilization of endogenous progenitor cells in the treatment of MI. The model of MI sought to simulate the condition of temporary coronary occlusion with reestablishment of coronary flow within less than 2 hrs and cell transplantation *via* the re-opened coronary or direct myocardial injection. We transplanted SDF-1 expressing transgenic EPCs to mobilize endogenous progenitor cells and augment the number of newly formed vessel structures as well as the expression of SDF-1 in the infarcted areas. A lentiviral construct encoding SDF-1 was chosen for gene delivery with control experiments demonstrating an EPC infection rate of 90% before transplantation. SDF-1-ELISA of the supernatant in cell cultures also showed highly significant elevation of SDF-1 production. SDF-1 relies on a concentration gradient to attract progenitor cells to the site of injury in this study, accordingly systemic SDF-1 concentration in peripheral blood samples from defined time points after cell transplantation did not differ between groups (data not shown). SDF-1 is also thought to facilitate migration of cells across the endothelial barrier therefore supporting endogenous circulating stem cell populations in reaching the infarcted myocardium [31].

Table 1 summarizes the results of the present study displaying changes relative to controls and levels of significance 8 weeks after cell transplantation. Temporary myocardial ischaemia and reperfusion led to a significant impairment of LV function in the control groups. Cell transplantation especially with intramyocardial application and in combination with SDF-1 expressing EPC led to an increase of LV function. This correlates best with the findings for neovascularization, which was also most pronounced in the groups performed with intramyocardial application as well as SDF-1 expressing EPC. Looking at remodelling parameters the infarcted areas in the groups of intramyocardial cell injection contained less collagen but more blood vessels. Because this effect was independent of SDF-1 expression it might be associated with a different retention of injected EPC *via* an intramyocardial route. However the absolute number of residual and remaining stem cells could not be determined therefore this hypothesis would remain speculative. Another parameter of remodelling was apoptosis which was clearly stimulated by SDF-1 expression. Although the study was not designed to describe the cell population undergoing apoptosis it has been described elsewhere that anti-apoptotic effects are desirable features of stem cell therapy [32]. On the other hand the SDF-1 expressing implanted cells induced proliferation in the infarcted area. Again, the type of proliferating cells was not identified but studies performed with indirect methods to increase SDF-1 secretion demonstrated an increase in c-Kit(+) and CD34(+) stem cells suggesting a certain portion of cardiac progenitor cells and endogenous bone marrow-derived cells [33–35]. At the time-point of experiment termination both groups of SDF-1 expressing EPC were associated with lower neutrophils but higher monocyte content in the infarcted territory. In agreement to other studies, a part of the monocyte population might represent migrated bone marrow derived MCs.

**Table 1:** Summarization of histological and functional results of the present study displaying changes relative to controls and levels of significance 8 weeks after cell transplantation

	Macrocytes	Monocytes	Apoptosis	Proliferation	Collagen	Angio-genesis	Echo (FS in %)	LVDP
EPC i.c.	**=**	**=**	**=**	**=**	**=**	(↑)	(+2.9%)	**=**
EPC i.m.	**=**	↑	**=**	**=**	↓	↑↑↑	(+7.4%)	↑
EPC/SDF1 i.c.	↓	(↑)	↑↑	↑	**=**	↑	(+6.4%)	**=**
EPC/SDF1 i.m.	↓	↑	↑↑↑	(↑)	↓	↑↑↑	+ 8.9%	↑

↑ or ↓:*P* < 0.05; ↑↑ or ↓↓:*P* < 0.01; ↑↑↑ or ↓↓↓: *P* < 0.001; (↑): strong tendency but non significant; =: no relevant changes.

The other aim of our study was to compare different application methods. Therefore, we created a novel intracarotid rodent balloon catheter system for an intracoronary cell application. Although the intramyocardial injection appeared to be more efficient in the groups performed with EPCs only the additional expression of SDF-1 somewhat compensated for that. One possible mechanism explaining the different results of intracoronary cell application might be that SDF-1 facilitated transendothelial migration for some of the remaining transplanted as well as endogenous cells as demonstrated through a CXCR4 receptor mediated mechanism elsewhere [36].

Limitations of the study lie in particular in the infarction model chosen that led to only relatively moderate degrees of MI, even in the group most affected the mean fractional shortening was still 33.3% or had - compared to baseline - decreased by 14.9%. Although this model of infarction sought to emulate clinical circumstances with infarction and early reperfusion the moderate infarction size might have blurred the differences between the EPC groups. This and the sample size of 8–12 animals per group also meant that although obvious trends of improvement were seen they did not reach statistical significance in all groups. An additional limitation is the single time-point at which the experiment was ended, the persistent level of inflammatory response and proliferation/apoptosis detected in some groups could be interpreted as continuing remodelling that has not yet reached its conclusion. Although we postulate that SDF-1 supports mobilization of endogenous stem cells from bone marrow, an effect that has also been published elsewhere [31], the amount of mobilized stem cells was not quantified in the present study. As with most small animal models the potential effect of concomitant pharmacotherapy used in clinical medicine has not been taken into consideration. It has to be further acknowledged that the endpoints of our study were rather functional parameters than clinical parameters such as LV function. A statistically significant increase of a functional parameter would not necessarily lead to a clinical benefit.

## Conclusion

Infection of EPC with SDF-1 is feasible and leads to SDF-1 secretion. LV-SDF-1-EPC can be transplanted *via* coronary route or through intramyocardial injection and persist in tissue. Although an intramyocardial transplantation of EPC seems to be more successful than intracoronary injection, the addition of SDF-1 expression seems to partially compensate for that effect. SDF-1 expression changes the tissue response and it remains to be determined which of these responses are desirable for improvement of LV function and remodelling. Additional studies are required to evaluate the effect of transgenic SDF-1 expression through stem cells in respect to infarct size, time course, animal model and concomitant medication or chemokine application.
