# Erratum

**DOI:** 10.1111/jcmm.12126

**Published:** 2013-12-23

**Authors:** A Schuh, A Kroh, S Konschalla

**Affiliations:** Myocardial regeneration by transplantation of modified endothelial progenitor cells

In 1, the eighth author's name should be Tolga Taha Sönmez (first name, middle name, last name).

The authors wish to apologise for any misunderstanding or inconvenience caused.
